# How flies are flirting on the fly

**DOI:** 10.1186/s12915-016-0342-6

**Published:** 2017-02-14

**Authors:** Courtney Eichorn, Michael Hrabar, Emma C. Van Ryn, Bekka S. Brodie, Adam J. Blake, Gerhard Gries

**Affiliations:** Department of Biological Sciences, Simon Fraser University, 8888 University Drive, Burnaby, British Columbia V5A 1S6 Canada

**Keywords:** *Lucilia sericata*, Mate location, Visual mate signals, Wing flashes, Flicker fusion frequency

## Abstract

**Background:**

Flies have some of the most elaborate visual systems in the Insecta, often featuring large, sexually dimorphic eyes with specialized “bright zones” that may have a functional role during mate-seeking behavior. The fast visual system of flies is considered to be an adaptation in support of their advanced flight abilities. Here, we show that the immense processing speed of the flies’ photoreceptors plays a crucial role in mate recognition.

**Results:**

Video-recording wing movements of abdomen-mounted common green bottle flies, *Lucilia sericata*, under direct light at 15,000 frames per second revealed that wing movements produce a single, reflected light flash per wing beat. Such light flashes were not evident when we video-recorded wing movements under diffuse light. Males of *L. sericata* are strongly attracted to wing flash frequencies of 178 Hz, which are characteristic of free-flying young females (prospective mates), significantly more than to 212, 235, or 266 Hz, characteristic of young males, old females, and old males, respectively. In the absence of phenotypic traits of female flies, and when given a choice between light emitting diodes that emitted either constant light or light pulsed at a frequency of 110, 178, 250, or 290 Hz, males show a strong preference for the 178-Hz pulsed light, which most closely approximates the wing beat frequency of prospective mates.

**Conclusions:**

We describe a previously unrecognized visual mate recognition system in *L. sericata*. The system depends upon the sex- and age-specific frequencies of light flashes reflecting off moving wings, and the ability of male flies to distinguish between the frequency of light flashes produced by rival males and prospective mates. Our findings imply that insect photoreceptors with fast processing speed may not only support agile flight with advanced maneuverability but may also play a supreme role in mate recognition. The low mating propensity of *L. sericata* males on cloudy days, when light flashes from the wings of flying females are absent, seems to indicate that these flies synchronize sexual communication with environmental conditions that optimize the conspicuousness of their communication signals, as predicted by sensory drive theory.

**Electronic supplementary material:**

The online version of this article (doi:10.1186/s12915-016-0342-6) contains supplementary material, which is available to authorized users.

## Background

Mate-seeking animals typically rely on sexual communication signals that facilitate mate encounters [1]. While these signals can be diverse (olfactory, visual, acoustic, vibratory, tactile) and may involve multiple sensory modalities [2], certain taxonomic groups use specific primary modalities of communication. Flies (Diptera) have some of the most advanced visual systems in the Insecta [3], often featuring large, sexually dimorphic eyes with specialized “bright zones” [4–6] that may have a functional role during mate-seeking behavior. The fast visual system of flies [7] is considered an adaptation that evolved in support of their advanced flight abilities [3], demanding superb visual acuity to gauge distance traveled and to avoid collisions [3]. Blow flies (Calliphoridae) exploit visual cues when they forage [8], seek oviposition resources [9], or pursue prospective mates [10]. Males have larger eyes than females [6], suggesting that females send and males perceive the visual signals or cues. Occupying vantage points in their territories, males survey rapid fly-bys of females and males, and then decide whether to fend off rival males or pursue prospective female mates.

The design and processing speed of the flies’ compound eyes allow us to infer functional linkage [11] of the visual communication signals that females send and males perceive. Blow flies possess rapid temporal visual discrimination; the flicker fusion threshold (the frequency at which blinking lights are perceived to be constant [12]) of the common green bottle fly *Lucilia sericata* (Diptera: Calliphoridae) exceeds 180 Hz [13], and may double at temperatures above 30 °C, as shown for the blue bottle fly *Calliphora vicina* [14], enabling these flies to resolve extremely fast or brief visual stimuli. This ability has been interpreted as an adaptation to support the flies’ advanced flight and collision-avoidance capabilities [3]. If this adaptation were to function also in mate recognition, one would expect an extremely fast and specific visual signal produced by females. We show that *L. sericata* males distinguish between the rates of light flashes reflected off the moving wings of female and male flies, and are most strongly attracted to flash frequencies characteristic of young females that are prospective mates.

## Results

**Fig. 1:**
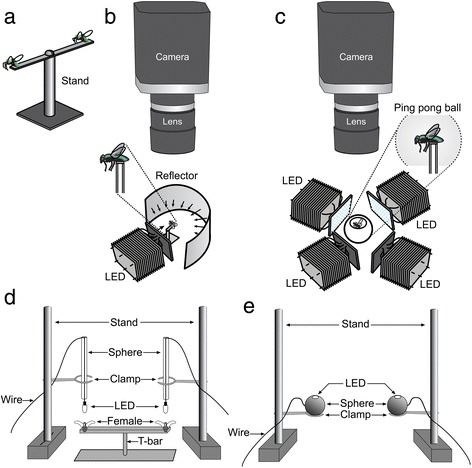
Graphical illustrations of experimental designs. **a** T-bar (vertical stand: 3.5 cm tall; horizontal bar: 7.5 cm long) with two *Lucilia sericata* flies mounted on their abdominal ventrum to leave their legs without support and thus induce a wing fanning response. **b**, **c** Set-up for high-speed video recordings of an abdomen-mounted, wing-fanning fly under direct light (**b**) or diffuse light (**c**) provided by one or four 100-watt cool light-emitting diodes (LEDs; see Methods for further details). **d** Mounted LEDs producing pulsed or constant light directed on to the immobilized wings of paired abdomen-mounted flies. **e** Black acrylic sphere holding a white-light LED directed upward; sanding the lens ensured that the emitted light was visible to flies from many viewing angles rather than from the narrow viewing angle that the lens otherwise creates

**Fig. 2:**
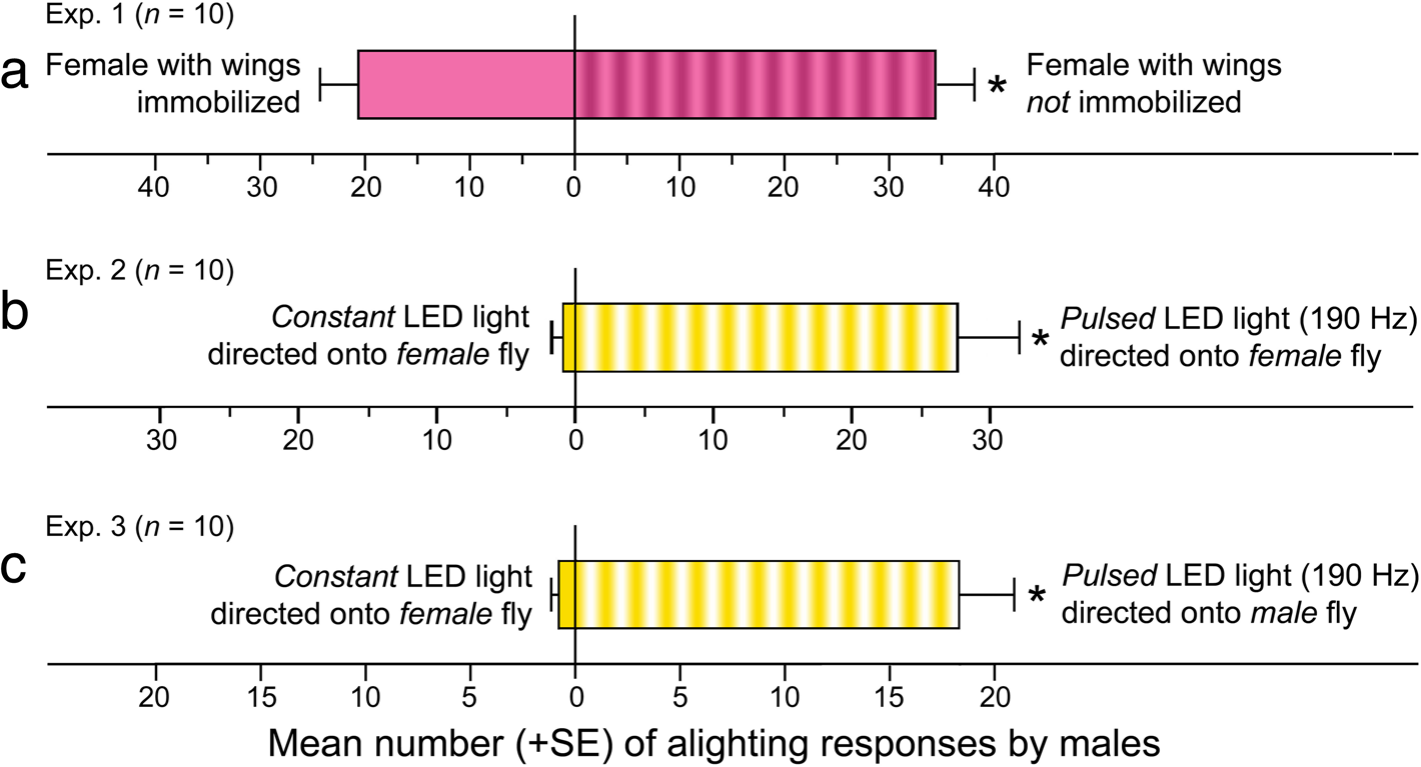
Alighting responses by *Lucilia sericata* males in two-choice experiments. **a**–**c** Number of alighting responses on or near paired mounted flies (Fig. 1a) that were able, or not, to move their wings (**a**), or that could not move their wings and were illuminated by pulsed or constant light (Fig. 1d) (**b**, **c**). In each experiment, an asterisk (*) indicates a significant preference for a test stimulus (*t-*test; *P* < 0.05)

Our search for visual mate recognition cues took into account that mate-seeking males pursue flying females. To test whether wing movement of females affects mate recognition by males, we mounted two live females side by side (Fig. 1a), immobilized the wings of one randomly assigned female and recorded the number of alighting responses by males on or near each female. Significantly more alighting responses by males on or near females that could move their wings than on females that could not (mean ± SE: 34.10 ± 3.76 vs. 20.60 ± 3.79; n = 10, *t* = –4.43, t crit. two-tail = 2.26, *P* = 0.002; Fig. 2a; Additional file 1: Data S1) revealed that wing movements by females contribute to mate selection by males.

**Fig. 3:**
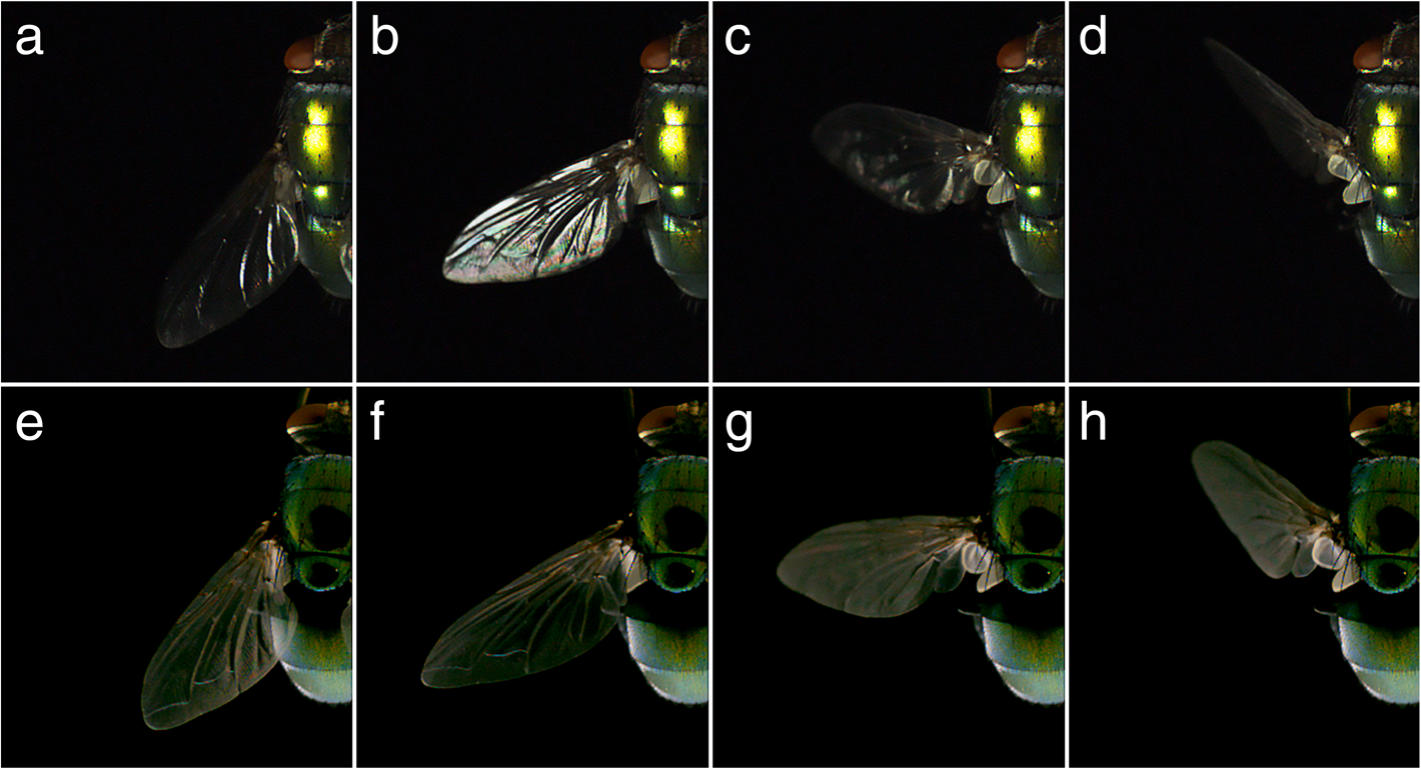
Effect of direct or diffuse illumination on the occurrence of wing-reflected light flashes. Single-frame photographs of fanning wings of abdomen-mounted *Lucilia sericata* females taken from high speed video recordings (15,000 frames per second) under direct light (**a**–**d**) or under diffuse light (**e**–**h**). **a**–**d** Photographs in the upper row reveal changes in the intensity of light reflected off the wing as it rotates during wing fanning, thus causing a flashing light effect in **b**; **e**–**h** Photographs in the lower row fail to reveal any flashing light effect.

**Fig. 4:**
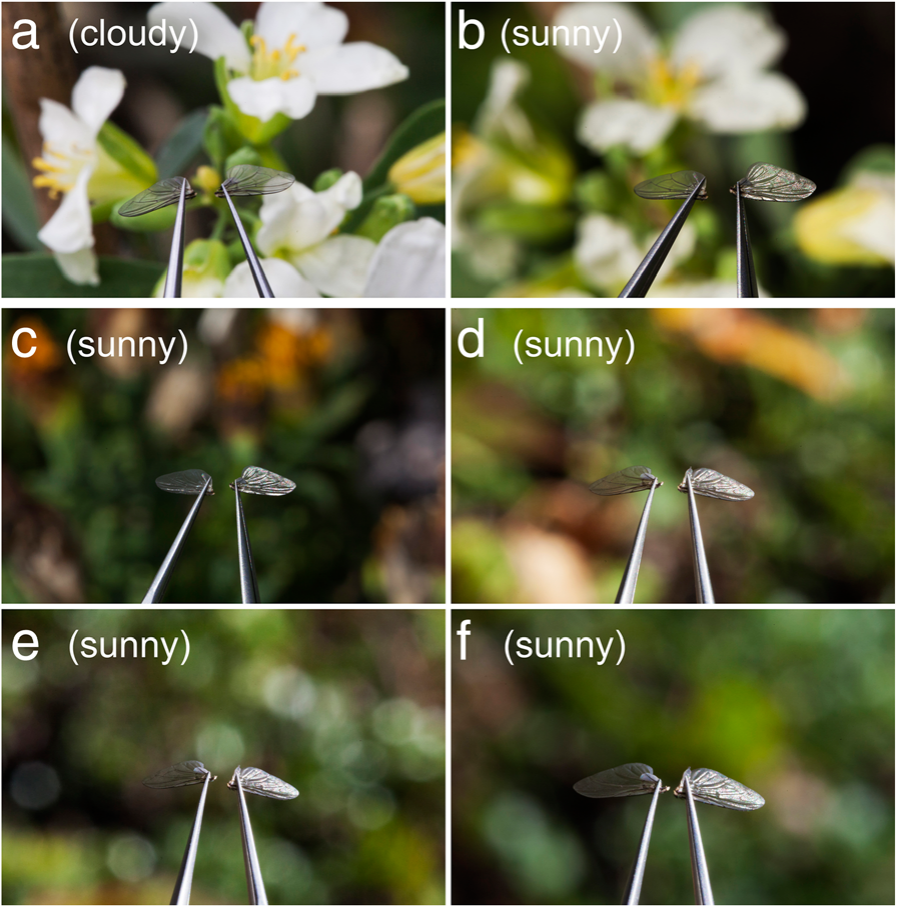
Photographs of *Lucilia sericata* wings mounted on hemostatic clamps and exposed to diffuse sunlight (**a**) and direct sunlight (**b**-**f**) on a day with periods of sunshine and clouds. In sub-panels **b**–**f**, note the bright sunlight reflected off the right wing in each pair

To visualize optic effects associated with moving wings of *L. sericata* females, we video-recorded the wing movement of abdomen-mounted flies under direct light at 15,000 frames per second (Fig. 1b), and found that wing movements produce a single, reflected, light flash per wing beat (Fig. 3a–d). Such light flashes (strong light reflections) were not evident (Fig. 3e–h) when we video-recorded wing movements under diffuse light (Fig. 1c), or when we took photographs of *L. sericata* wings outdoors under a cloudy sky (Fig. 4a). Butterflies and blow flies ([15], this study) exhibit low mating propensity on overcast days when otherwise direct illumination from the sun becomes diffuse [11, 16], and thus reduces flash effects [17]. These observations support the hypothesis that light reflected from the wings produces beacons that contribute to mate recognition.

We tested this hypothesis using two approaches. First, we mounted two live female flies on an aluminum T-bar (Fig. 1d), immobilized their wings, and illuminated each female by a light emitting diode (LED) (Additional file 2: Figure S1a), one that produced light pulses at 190 Hz approximating the wing flash frequency of a flying female, and the other that produced constant light at the same intensity. Second, we isolated the pulsed-light effects from phenotypic traits of female flies by mounting one live female fly and one live male fly side by side (Fig. 1d), immobilizing their wings and illuminating the wings of the male by the 190-Hz light pulses while keeping the wings of the paired female under constant illumination. In both experiments, we placed the T-bar with the two mounted flies into a bioassay cage containing 50 male flies and recorded the numbers of alighting responses on either fly in each pair. In both experiments, the female or male fly exposed to pulsed light (190 Hz) received many more alighting responses (mean ± SE) by males than did the fly illuminated by constant light (Exp. 2: 27.8 ± 4.32 vs. 0.9 ± 0.31; n = 10, t crit. two-tail = 2.26, *t* = 6.44, *P <* 0.001; Exp. 3: 18.1 ± 2.98 vs. 0.8 ± 0.29; n = 10, t crit. two-tail = 2.26, *t* = 5.54, *P* < 0.001; Figs. 2b, c; Additional file 1: Data S1).

**Fig. 5:**
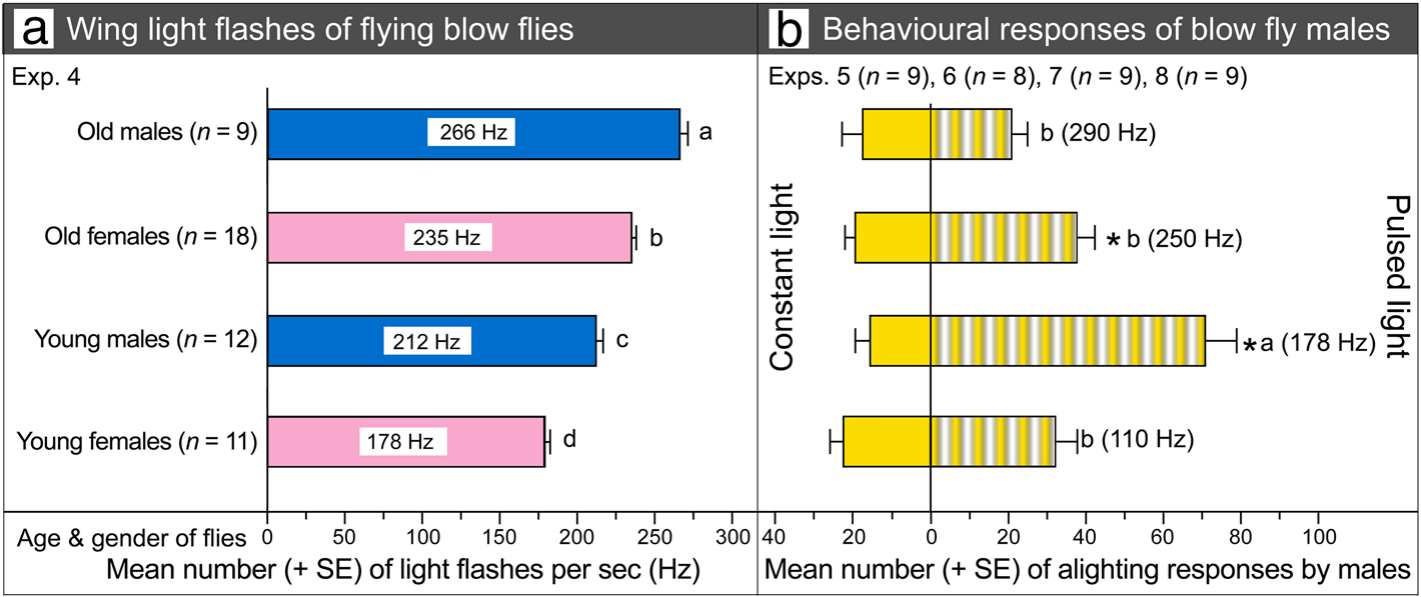
Effect of fly sex and age on the frequency (Hz) of wing-reflected light flashes and their effects on attraction of males. **a** Numbers of light flashes per second reflected off the wings of free-flying 2-day-old (young) and 7-day-old (old) female and male *Lucilia sericata; n* indicates the number of flies analyzed in each category; different letter superscript on bars indicate significant differences in light flash frequencies based on the age and sex of flies (ANOVA followed by the Tukey test for comparisons of means). **b** Alighting responses by *L. sericata* males on paired black acrylic spheres (Fig. 1e) each holding a white-light LED that emitted either constant light or light pulsed at a frequency of 290 Hz (Exp. 5), 250 Hz (Exp. 6), 178 Hz (Exp. 7), or 110 Hz (Exp. 8); the asterisk in Experiments 6 and 7 indicates a significant preference for the specific pulsed light stimulus (*t*-test); different letter superscripts on bars indicate significant differences in alighting responses based on the frequency of pulsed light (one-way ANOVA followed by the Tukey test for comparisons of means)



While these results support the hypothesis that light pulses contribute to mate recognition, such signals or cues can be functional in mate recognition only if they differ in frequency by sex and age of free-flying individuals. Only then would a male fly be able to distinguish between rival males and prospective mates traversing his territory. We tested this hypothesis by filming young and old male and female flies in free flight, using a Phantom Miro 3 high-speed camera at a rate of 15,325 frames per sec. For each recording event, we placed 50 young or 50 old male or female flies into a wire mesh cage fitted with a cool-white LED. Following each recording, which typically captured 1–6 flies in free flight, we proceeded to the next recording with a new set of flies in another cage. Analyzing the video-recorded data files (e.g.; Additional file 3: Video S1; Additional file 4: Video S2), we found differences in the frequency of light flashes reflected off the wings of free-flying young and old females and young and old males (one-way ANOVA; *F*_*3,46*_ = 96.22, *P* < 0.001; Additional file 1: Data S1). Young females had a mean (± SE) flash frequency of 178.72 Hz (±2.86 Hz), which was significantly lower than that of young males (212.0 ± 4.18 Hz), old females (235.08 ± 2.58 Hz), and old males (265.78 ± 4.53 Hz) (Fig. 5a). Because *L. sericata* males seek young (2- to 4-day-old) females as prospective mates [18], the slower wing flash frequency from young females than from young males, or older individuals of either sex, could be a phenotypic trait of reproductively capable females.

**Additional file 3: Video S1.** High-speed video recording (15,325 frames per second; see Methods for details) of a green bottle fly, *Lucilia sericata*, filmed in the laboratory within a wire mesh cage illuminated by a cool-white LED (see Additional file 2: Figure S1a). Note the bright light flashes reflecting off the wings. (MP4 4411 kb)




**Additional file 4: Video S2.** High-speed video recording (15,325 frames per second; see Methodsfor details) of a green bottle fly, *Lucilia sericata*, filmed in the laboratory within a wire mesh cage illuminated by a cool-white LED (see Additional file 2: Figure S1a). Note again the bright light flashes reflecting off the wings. (MP4 2159 kb)


If the lower wing flash frequency of young females is the key signal or cue for mate recognition by mate-seeking males, one would expect males to respond to this frequency even in the absence of live females and to distinguish between flash frequencies that are true mate cues (178 Hz; see above) and those that are not. By eliminating females from the experimental design, we isolated the light flash effect as the test variable. We ran four experiments in parallel and recorded alighting responses by males on paired black acrylic spheres (Fig. 1e) instead of paired mounted female flies. Each sphere in each pair held a white-light LED, one emitting constant light, the other emitting light pulses at a frequency of 290, 250, 178, or 110 Hz. The 250-Hz pulses represent light flashes produced by flying old females and males (see above). We selected 290- and 110-Hz pulses to test the response of males to pulse frequencies that are well above or below the wing flash frequencies produced by flying conspecifics.

Males did not respond to light pulsed at 290 or 110 Hz in greater numbers than to constant light (Fig. 5b). Spheres holding an LED emitting 250-Hz light pulses received twice as many alighting responses by males than did the spheres with an LED emitting constant light (Exp. 6: 37.5 ± 4.97 vs. 19.37 ± 2.54; n = 8, t crit. two-tail = 2.37, *t* = 4.59, *P =* 0.003; Fig. 5b). Spheres with an LED emitting the 178-Hz pulses indicative of prospective mates received not only 4.2 times more alighting responses by males than did the paired spheres with an LED emitting constant light (Exp. 7: 70.3 ± 8.93 vs. 15.67 ± 3.08; n = 9, t crit. two-tail = 2.31, *t* = 7.21, *P <* 0.001) (Fig. 5b), but they also received significantly more alighting responses than any of the other light-pulsing LEDs (one-way ANOVA; F_3,31_ = 13.55, *P* < 0.001; Additional file 1: Data S1). Together, these results reveal that *L. sericata* males do not simply prefer pulsed light to a constant light, but also prefer pulsed light that occurs at frequencies corresponding to the wing flash frequency of young females that are prospective mates.

To ascertain that *L. sericata* wing flashes are clearly visible even in diverse natural habitats and thus can indeed function in mate recognition, we took photographs of *L. sericata* wings exposed to direct sunlight in outdoor settings, and video-recorded at high speed free flying *L. sericata* exposed to direct sunlight. Sunlight-reflecting stationary (mounted) wings produced bright visible cues (Fig. 4b–f), appearing 2–3 times brighter than the paired wing positioned at an angle not conducive to reflecting the sunlight (Additional file 2: Figure S1b). In laboratory measurements, the spectral power distribution of light reflected by *L. sericata* wings closely resembled that of the incident light (Additional file 2: Figure S1c). Moreover, wings of *L. sericata* females free flying in an outdoor setting produced repeated flashes of light that contrasted well even against a complex background of plant foliage (Additional file 5: Video S3; Additional file 6: Video S4).



**Additional file 5: Video S3.** High-speed (15,000 frames per second) video recording (see Methods for details) of a female green bottle fly, *Lucilia sericata*, exiting a pipette and taking flight against a plant-covered slope illuminated by direct sunlight. Note how well the light flashes reflecting off her moving wings contrast against the background. (MP4 6067 kb)




**Additional file 6: Video S4.** High-speed (7000 frames per second) video recording (see Methods for details) of a female green bottle fly, *Lucilia sericata*, traversing a plant-covered slope illuminated by direct sunlight. Note again how well the light flashes reflecting off her moving wings contrast against the background, even when she is moving out of focus. (MP4 6615 kb)


## Discussion

Moving wings are thought to mediate long-range detection of potential mates in some butterflies and damselflies [17, 19, 20]. In these insects, light flash effects coupled with other visual effects of moving wings such as iridescence, UV, and polarized light reflections are hypothesized to contribute to mate recognition. In our study, we have decoupled light flash effects from other effects of moving wings, demonstrating that male flies respond to the light flashes per se when they seek prospective mates. By showing that *L. sericata* males respond to LED light pulses in the absence of females, we provide evidence that the frequency of pulsed light is the key mate recognition cue in *L. sericata* and that this cue is independent of structural and color characteristics of female wings.

Unlike static signals or cues, flashing signals affect improved visibility [19, 21]. This is evident, for example, in Morpho butterflies whose wings produce light flashes that are reported to be visible from low-flying aircraft [22] and in *Heliconius* butterflies whose wings produce polarized light flashes that stand out in complex forest habitats [20]. Compared to these butterfly light flashes, the light flashes produced by *L. sericata* wings are not only highly visible in direct sunlight (Fig. 4; Additional file 5: Video S3; Additional file 6: Video S4), they are also very rich in information content. Divergent flash rates produced by young and old females (Fig. 5a), and the males’ ability to “read” these rates (Fig. 5b), allow for the conveyance of information that enables informed mate assessment. Exploitation of “rate-based” signals or cues may, in fact, be commonplace in the Insecta. For example, rates of flashing light signals produced by bioluminescent fireflies likely convey important information about mate suitability [12, 23].

Sensory perception of light flashes produced by moving wings seems to be facilitated by the functional design, neural circuitry, and processing speed found in the sexually dimorphic compound eyes of several species of flies. For example, males but not females of the blow fly *Chrysomya megacephala* and the hover fly *Eristalis tenax* have large ommatidial facets in their dorsal frontal eye region [4, 5] that form a “bright zone” believed to be capable of increased light capture. This bright zone is not linked to enhanced resolution [24], but is deemed to allow males to search for females at low light or from great distances in bright light [24]. We hypothesize that this bright zone may also help males detect the flashing lights of prospective mates. Moreover, the fast photoreceptors of calliphorid flies [25] may not only underlie adaptations of a visual system that has evolved to support advanced flight and collision-avoidance capabilities [3], but also may enable a superior function in mate recognition. The temporal encoding ability of *L. sericata* males was amply sufficient to distinguish between light flash frequencies of prospective mates (178 Hz) and rival males (250 Hz) (Fig. 5b). While numerical competence is known for mammals [26, 27], amphibians [28], birds [29], fish [25], and some invertebrates such as ants [30], the numerical recognition and signal-processing speed exhibited by *L. sericata* males seem to top currently known records.

The logical framework offered by the sensory drive theory [11] predicts functional links between signal design and presentation such that signal conspicuousness is maximized relative to background noise or environmental conditions. Our data on *L. sericata* are in complete agreement with this prediction. Unlike the iridescent light flashes produced by *Hypolimnas bolina* butterfly wings [17] that are most conspicuous only from a narrow perspective [17], the wing flashes produced by flying *L. sericata* females are visible beacons (Fig. 4; Additional file 5: Video S3; Additional file 6: Video S4) that are detectable from all directions, allowing a territorial male fly to rapidly notice a female irrespective of her flight trajectory, particularly when he is perching at a vantage point that optimizes contrast between fly flash signals and background. Remarkably, the flash frequency is so informative that it allows the territorial male to distinguish between old and young females, and to pursue primarily young females that are preferred mates. Furthermore, the low mating propensity of *L. sericata* on overcast days, when diffuse sunlight renders light reflections off wings inconspicuous (Fig. 4a), appears to show that these flies time their sexual communication and mating activities in accordance with environmental conditions that optimize the conspicuousness of their sexual communication signals.

## Conclusion

In conclusion, we describe a previously unidentified visual mate recognition system in the common green bottle fly. The system depends upon both the sex- and age-specific frequencies of light flashes reflecting off moving wings, and the ability of male flies to distinguish between the frequency of light flashes produced by rival males and prospective mates. Our findings imply that insect photoreceptors with fast processing speed may not only support agile flight with advanced maneuverability but may also play a supreme role in mate recognition. With emerging evidence that light flash mate cues also occur in other insects (unpublished data), there may be an opportunity for optimizing light traps for capture of specific nuisance insects in urban and industrial settings.

## Methods

### Experimental insects

We reared *L. sericata* in the insectary at Simon Fraser University, starting a new colony with field-collected wild flies every 12 months. We cold-sedated flies within 24 h following eclosion, separated them by sex, and kept them in groups of 50 males or 50 females in separate wire mesh cages (45 × 45 × 45 cm; BioQuip®, Compton, CA, USA) under a L16:D8 photoperiod, 30–40% relative humidity, and 23–25 °C. We provisioned flies with water, milk powder, sugar, and liver ad libitum and used 2- to 7-day-old flies in bioassays.

### Responses by males to mounted females, one able to wing-fan, the other with wings glued to her body

For each replicate of Experiment 1 (n = 10), we CO_2_-sedated two live female flies for 30 s, and then mounted them with cyanoacrylate adhesive on their abdominal ventrum, at opposite ends of a 7.5-cm-long aluminum T-bar (Fig. 1a). We applied a small amount of cyanoacrylate to the wing base of one randomly assigned female to immobilize her wings, and applied the same amount of adhesive to the abdomen of the other female, allowing her wings to move freely. We placed the T-bar with the two females in a wire mesh bioassay cage (45 × 45 × 45 cm; BioQuip®) containing 50 male flies. The cage was illuminated from above with a full spectrum light source (two horizontal fluorescent bulbs: Philips, plant & aquarium (40 W); Sylvania, Daylight Deluxe (40 W)) (Additional file 2: Figure S1a). To minimize light reflection, we covered the metal cage floor and T-bar stand with SunWorks® black construction paper and black velvet (Suzhou Joytex International Co. Ltd., Jiangsu, China), respectively. During each 40-min bioassay, we recorded the number of alighting events by males on or near a female followed by physical contact with her. We analyzed the mean numbers of alighting responses by males on females with wings either mobile or immobilized by a paired two sample for means *t*-test.

### Do moving wings produce flashes of reflected light under point source illumination?

We recorded the wing movement of abdomen-mounted male and female flies (Fig. 1b) in slow motion using a Phantom Miro 4 camera (Vision Research, Wayne, NJ, USA), recording at 15,000 frames per second, a 512 × 512 pixel resolution, and a 20-μs exposure time. To illuminate the mounted fly, we used a white 100-watt LED (6500 K; Zongshan Ltd., Guangdong, China) mounted to a computer CPU heat sink for cooling (Thermaltake Heatpipe, Thermaltake Technology Co. Ltd, Taipei, Taiwan), and powered via a 32 V 5A stabilized, adjustable DC power supply (Gopher Technologies, Yantian, Fenggang, Dongguan, Guangdong, China).

### Do moving wings produce flashes of reflected light under diffuse illumination?

We used the same high-speed video technology as described above, except that we exposed the mounted fly to diffuse instead of point source light. We placed the fly inside a ping pong ball “diffuser” (Fig. 1c) and illuminated it by four cool white 100-watt LEDs (see above).

### Responses by males to paired mounted females, both with their wings immobilized but one with pulsed light reflecting off her wings

For each replicate (n = 13) of Experiment 2, we mounted two live female flies on an aluminum T-bar (Fig. 1d) and immobilized the wings of each female with cyanoacrylate adhesive. We illuminated one randomly assigned female from above by a light emitting diode (LED, Optek Technology Inc., Carrollton, Texas 75006, USA) (Additional file 2: Figure S1a) mounted 3 cm above the female (Fig. 1d) and which produced 5-Volt, white-light pulses at a frequency of 190 Hz and a duty cycle of 3%. The pulse frequency of 190 Hz was approximately mid-way between the light-flash frequencies of flying 2-day-old female and male flies. We illuminated the control female by a second LED of the same type that produced constant light.

We considered two alternative approaches to the illumination design of this experiment. We could have set the pulsed-light LED and the constant-light LED to deliver either equal total light intensity (root mean squared) or equal maximum light intensity (peak voltage). We chose the latter (conservative) approach because, at a 3% duty cycle (“on” vs. “off” ratio), the pulsed-light LED delivers only about 3% of the total light that the constant-light LED delivers. Thus, to the human eye, the pulsed-light LED appears as a constant dim light, whereas the constant-light LED appears as a constant bright light; to fly photoreceptors, in contrast, the pulsed-light LED appears as an intermittent (pulsing) light with a light intensity matching that of the constant-light LED.

For each replicate, we placed the T-bar with the two females into a wire mesh bioassay cage containing 50 male flies. During 40 min in each replicate, we recorded the numbers of alighting responses by these 50 male flies on each female, and analyzed the mean numbers of alighting responses by a paired two sample for means *t*-test.

### Responses by males to paired male and female flies, both with their wings immobilized, and pulsed light reflecting off the male’s wings

In each replicate of Experiment 3 (n = 10), we mounted one live female fly and one live male fly 7 cm apart on an aluminum T-bar (Fig. 1d), and immobilized the wings of each fly with cyanoacrylate adhesive. We illuminated the male from above by an LED (Fig. 1d) that produced 5-Volt, white-light pulses at a frequency of 190 Hz and a duty cycle of 3%. We illuminated the female by a second LED of the same type that produced constant light at equal maximum intensity as the first LED. During 40 min in each replicate, we recorded the number of alighting responses by 50 males on the mounted male and female fly, analyzing the mean number of alighting responses on the male and female by a *t*-test.

### Light flash frequencies associated with age and sex of flying individuals

The objective of Experiment 4 was to determine whether the numbers of light flashes reflected off the wings of free-flying flies differ in accordance with age or sex. We filmed 2-day-old (young) and 7-day-old (old) male and female flies in free flight using a Phantom Miro 3 high-speed camera (Vision Research) at a rate of 15,325 frames per second and a 34-μs exposure time imaged through a Canon 100-mm f2.8 L macro lens (Canon Canada Inc., Vancouver, BC V6C-3 J1, Canada) fitted to a 36-mm extension tube. For each recording event, we placed 50 young or 50 old male or female flies into a wire mesh cage (45 × 45 × 45 cm) housing a 100-Watt (approximately 9000 Lumen), cool-white (5000–6000 Kelvin) LED, which was driven by a 32-Volt switching-power supply (model CPS-3010, Gopher Technologies, Yantian, Fenggang Town, Dongguan, Guangdong, China). Once the camera and light were turned on, we lightly tapped the cage to induce take-off and flight by resting flies. In video-recorded data files, we counted the number of light flashes reflected within one second off the wings of free-flying young females (n = 11), young males (n = 12), old females (n = 18), and old males (n = 9), and analyzed light-flash frequencies of young and old females and of young and old males by one-way ANOVA followed by the Tukey test for comparisons of means.

### Ability of males to discriminate between LED-pulsed light of varying frequencies

To determine whether mate-seeking males can distinguish between different frequencies of pulsed light, parallel-run behavioral Experiments 5–8 (n = 9, 8, 9, and 9, respectively) tested alighting responses by males on paired black acrylic spheres (1.77 cm diameter; supplier unknown; Fig. 1e). We mounted the spheres on clamps 12 cm apart and 12 cm above the floor of the bioassay cage containing 50 male flies. A central hole (0.52 cm) in each sphere accommodated an upward pointing LED (Fig. 1e), the rounded lens of which was sanded down to be flush with the sphere’s surface. Sanding the lens ensured that the emitted light was visible to flies from many viewing angles rather than from the narrow viewing angle that the lens otherwise creates. By random assignment, one LED in each pair emitted constant light; the other emitted light pulsed at 290 Hz (Experiment 5), 250 Hz (Experiment 6), 178 Hz (Experiment 7), or 110 Hz (Experiment 8). We selected the frequencies of 290 Hz and 110 Hz to test the response of males to pulse frequencies that are well above or below the wing flash frequencies produced by flying common green bottle flies. In each of Experiments 5–8, we analyzed the mean numbers of alighting responses by males on paired spheres holding LEDs emitting constant light or pulsing light by a paired two sample for means *t*-test. We analyzed differences in alighting responses based on the frequency of pulsed light by one-way ANOVA followed by the Tukey test for comparisons of means.

### Visibility of light flashes reflected off the wings of free-flying flies video recorded outdoors under direct sunlight

To document the effect of sunlight reflecting off the wings of a free flying *L. sericata*, we took high-speed video recordings of flies traversing a south-facing slope with plant cover under direct, mid-day sun under a partially cloudy sky. For these recordings, we used a FASTCAM Mini AX200 type 900 K-M camera (Photron USA Inc., San Diego, CA 92126, USA) fitted with a Canon macro lens (100 mm; f2.8 L) at f5.6, capturing images at 15,000 frames per second, a 1/15000 exposure time, and a 768 × 512 pixel resolution.

### Effect of natural sunlight reflecting, or not, off *L. sericata* wings

We carefully removed wings from a 1-day-old female fly, mounted them on hemostatic clamps positioned by articulated holders (Noga Engineering Ltd., Shlomi 22832, Israel), and angled the wings such that the right wing, but not the left wing, reflected sunlight back toward the camera. We photographed the wings under cloudy conditions (Fig. 4a) and under sunny conditions (Fig. 4b–f), keeping the wings near minimum focus from the lens, with various distances to background foliage. We took the photographs with a Canon EOS 5D Mark II Full Frame DSLR camera fitted with a Canon EF 100 mm f2.8 L macro lens, using the following parameters: (1) 1/50 sec exposure, f29; (2) 1/160 sec exposure, f18; (3) 1/160 sec exposure, f22; (4) 1/80 sec exposure, f29; (5) 1/125 sec exposure, f29; and (6) 1/60 sec exposure, f 29. We converted RAW images to 16-bit uncompressed TIFF files using open-source RAW image-decoding software (DCRAW; [31]) in a manner that maintained pixel linearity. We then examined the images in ImageJ [32], separated the green color channel, manually selected wings, and graphed histograms of pixel values.

### Relative spectral power of light reflected off the wings of immobilized female flies exposed to a 100-watt white-light LED

We narrowed the field of view of a spectrometer (HR4000, Ocean Optics, USA) attached to a cosine corrector (CC-3-UV-S, Ocean Optics, USA), using a Gershun tube constructed of matte black construction paper. The tube extended 5 cm beyond the tip of the cosine corrector and had a 6-mm opening. We positioned decoupled female *L. sericata* wings as described above, keeping the wing and the aperture of the Gershun tube 2 cm apart. At this distance, the spectrometer’s field of view is limited to an 8-mm radius circle. Through this approach, we could maximize the field of view occupied by the wing. We took radiance spectra of (1) the illuminating 100-watt white-light LED, (2) the reflection from the matt black velvet background behind the wings, and (3) the reflectance of the wing oriented to reflect or (4) to not reflect, light towards the opening of the tube.

## Additional files

Additional file 1: Data S1. Raw data of all experiments and statistical analyses. (XLSX 116 kb)
Additional file 2: Figure S1. Relative spectral power distribution (SPD) of illumination devices and of light reflected off green bottle fly *Lucilia sericata* wings. **a** Relative SPD of fluorescent bulbs (dark grey) used to illuminate bioassay cages in Experiments 1–5, and of stimulus LEDs (light grey) used in Experiments 2–5; **b** Histogram of green pixel values from a *L. sericata* non-reflecting wing (dark grey; mean = 3490) and a reflecting wing (red; mean = 10,087) photographed in direct sunlight (Fig. 4d). The images in the legend show the portions of the wings used to calculate the histogram. **c** Relative SPD of (1) a 100-watt white-light LED, (2) the light cast back off *L. sericata* wings oriented to either reflect, or to not reflect, the incident LED light, and (3) the reflection from the background behind the wings; we normalized each of the spectra by its maximum spectral value. (JPG 429 kb)

## Abbreviations

ANOVA: Analysis of Variance
LEDs: Light emitting diodes.
