# Supplementary figures and images for: How flies are flirting on the fly
Source: BMC Biol. 2017 Feb 14;15:2. doi: 10.1186/s12915-016-0342-6 (PMC5307768; doi:10.1186/s12915-016-0342-6)

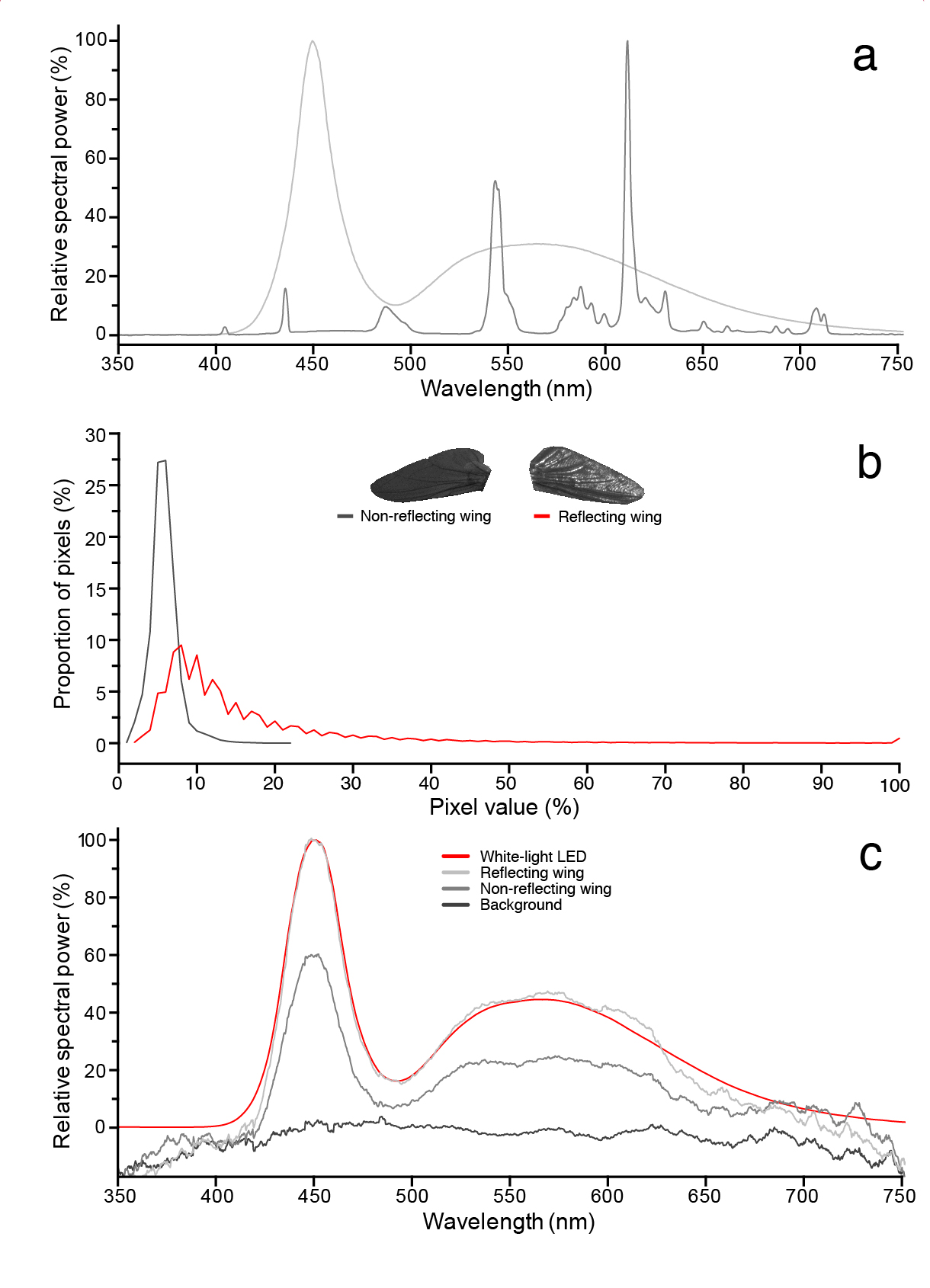

Supplement: Additional file 2: Figure S1. — Relative spectral power distribution (SPD) of illumination devices and of light reflected off green bottle fly Lucilia sericata wings. a Relative SPD of fluorescent bulbs (dark grey) used to illuminate bioassay cages in Experiments 1–5, and of stimulus LEDs (light grey) used in Experiments 2–5; b Histogram of green pixel values from a L. sericata non-reflecting wing (dark grey; mean = 3490) and a reflecting wing (red; mean = 10,087) photographed in direct sunlight (Fig. 4d). The images in the legend show the portions of the wings used to calculate the histogram. c Relative SPD of (1) a 100-watt white-light LED, (2) the light cast back off L. sericata wings oriented to either reflect, or to not reflect, the incident LED light, and (3) the reflection from the background behind the wings; we normalized each of the spectra by its maximum spectral value. (JPG 429 kb) [file 12915_2016_342_MOESM2_ESM.jpg]
